# Outbreak of Yellow Fever among Nonhuman Primates, Espirito Santo, Brazil, 2017

**DOI:** 10.3201/eid2312.170685

**Published:** 2017-12

**Authors:** Natália Coelho Couto de Azevedo Fernandes, Mariana Sequetin Cunha, Juliana Mariotti Guerra, Rodrigo Albergaria Réssio, Cinthya dos Santos Cirqueira, Silvia D’Andretta Iglezias, Júlia de Carvalho, Emerson L.L. Araujo, José Luiz Catão-Dias, Josué Díaz-Delgado

**Affiliations:** Instituto Adolfo Lutz, São Paulo, Brazil (N.C.C.A. Fernandes, M.S. Cunha, J.M. Guerra, R.A. Réssio, C.S. Cirqueira, S. D’Andretta Iglezias, J. de Carvalho, J. Díaz-Delgado);; Ministério da Saúde, Brasília, Brazil (E.L.L. Araujo);; Universidade de São Paulo, São Paulo (J.L. Catão-Dias, J. Díaz-Delgado)

**Keywords:** yellow fever, arboviruses, primates, epizootics, outbreak, pathology, virology, viruses, nonhuman primates, zoonoses, Brazil

## Abstract

In January 2017, a yellow fever outbreak occurred in Espirito Santo, Brazil, where human immunization coverage is low. Histologic, immunohistologic, and PCR examinations were performed for 22 deceased nonhuman New World primates; typical yellow fever features were found in 21. Diagnosis in nonhuman primates prompted early public health response.

Yellow fever is a reemerging, zoonotic, noncontagious viral hemorrhagic disease endemic to Africa and South America; outbreaks occasionally occur among human and nonhuman primates (*1*). It is caused by the yellow fever virus (family *Flaviviridae*, genus *Flavivirus*), which is carried by the vector mosquitoes *Haemagogus* and *Sabethes* (sylvatic cycle) and *Aedes aegypti* (urban cycle) (*2*).

Presumptive first reports of infection with yellow fever virus occurred ≈500 years ago (San Domingo, 1498; western Africa, 1585), and the first epidemics were recorded in the 17th century (Barbados, Cuba, and Mexico) (*3*–*5*). By the 18th century, epidemics were already deemed a threat for public health in the Old and New Worlds; transoceanic migrations played a major role in virus spread (*3*,*6*). After entering Brazil by the coast in the 17th century, the virus was gradually displaced to northwestern and midwestern areas. Since the 19th century, yellow fever outbreaks occurred in many cities in Brazil, until 1942, when the urban cycle was eradicated. By the late 1900s through the first decade of the 21st century, beginning in 1997, intense virus circulation extended from the Amazon region to the contiguous states of Goiás and Mato Grosso do Sul (central Brazil). During 2008–2009, a new outbreak was registered and the virus reached southern and southeast regions of the country (*1*). In 2017, an epizootic occurred in Espirito Santo state, Brazil, where yellow fever virus has not circulated in the past 50 years and human vaccination coverage is low. We performed diagnostics on a small cohort of New World nonhuman primates in this state.

## The Study

**Figure 1:**
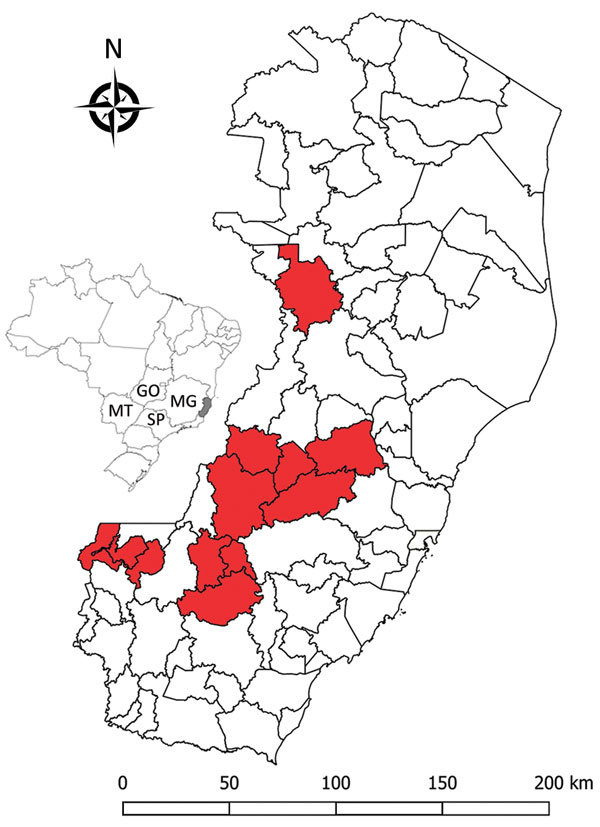
Locations (red shading) of nonhuman primates that died of yellow fever, Espirito Santo, Brazil, January 2017. Inset shows location of Espirito Santo (light gray shading) and 4 other states within Brazil. GO, Goias; MG, Minas Gerais; MT, Mato Grosso do Sul; SP, São Paulo.

In January 2017, an outbreak involving deaths of humans and New World nonhuman primates (hereafter nonhuman primates [NHPs]) spread from Minas Gerais state to Espirito Santo state. Espirito Santo is located in a forest along the Atlantic coast of southeastern Brazil and borders Minas Gerais, Rio de Janeiro, and Bahia states. The virus advanced through Atlantic forest fragments (*7*), areas previously considered not at risk for yellow fever virus transmission. We performed histopathologic analyses, immunohistochemical analyses (IHC), and PCRs on 22 NHPs that died early in the Espirito Santo outbreak (Figure 1).

Veterinarians trained by health authorities performed standardized necropsies under the yellow fever national surveillance program (*8*). Of 22 NHPs found dead, 2 were howler monkeys (*Alouatta* spp.) and 20 were NHPs not further identified. Liver, spleen, kidney, heart, and lung samples were collected and fixed in 10% neutral buffered formalin and processed for routine histopathology and in liquid nitrogen for RNA real-time reverse transcription quantitative PCR.

**Figure 2:**
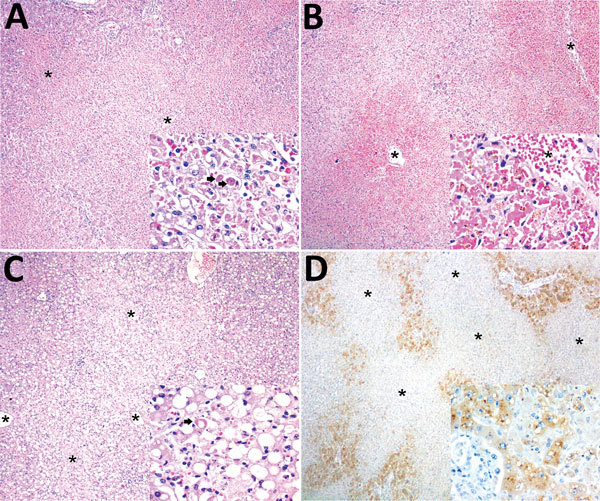
Histopathologic and immunohistochemical findings in the livers of neotropical nonhuman primates that died of yellow fever, Espirito Santo, Brazil, January 2017. Asterisks (*) indicate centrilobular veins. A) Midzonal and centrilobular bridging hepatocellular lytic necrosis. Original magnification ×40; hematoxylin and eosin (H&E) staining. Inset shows lytic hepatocellular necrosis with multiple Councilman-Rocha Lima (apoptotic) bodies (arrows). Original magnification ×400; H&E staining. B) Massive (diffuse) hepatocellular lytic necrosis with severe centrilobular and midzonal hemorrhage. Original magnification ×40; H&E staining. Inset shows prominent hepatocellular necrosis and dropout, and erythrocytes replace the hepatic cords (there is some artifactual formalin pigment [acid hematin] in necrotic hepatocytes). Original magnification ×400; H&E staining. C) Massive macrovacuolar steatosis. Inset shows massive macrovacuolar steatosis mingled with single-cell hepatocellular necrosis (arrow). Original magnification ×400; H&E staining. D) Positive immunolabeling confined to remaining periportal hepatocytes and terminal plate. Original magnification ×40; immunohistochemical staining for yellow fever virus. Inset shows positive granular, cytoplasmic immunolabeling for yellow fever virus antigen in periportal hepatocytes and terminal plate. Original magnification ×400; immunohistochemical staining for yellow fever virus.

Histologic examination indicated the following for all animals: zonal bridging (largely midzonal and centrilobular) or massive liver necrosis with Councilman bodies, varying degrees of macrovacuolar and microvacuolar steatosis, and pleocellular (mainly lymphohistiocytic) inflammatory infiltrates, accompanied by hemorrhage and hemosiderosis (Figure 2, panels A, B; Technical Appendix Table 1). Additional liver lesions were microabscesses (6), endothelial necrosis (5), oval cell hyperplasia (2), massive macrovacuolar steatosis with rare midzonal to random single-cell necrosis (1) (Figure 2, panel C), and fibrin microthrombi in sinusoids (1). Other consistent findings were splenic lymphoid depletion and follicular necrosis/lymphocytolysis (19), acute renal tubular necrosis with protein casts and hemoglobin casts (6), and multisystemic hemorrhage, more prominent in lungs (6).

For IHC, we tested liver tissue sections with an in-house primary polyclonal anti–yellow fever virus antibody (1:40,000, derived from hyperimmune mouse serum); signal amplification was achieved with a HiDef Detection HRP Polymer System (Cell Marque/Sigma-Aldrich, Rocklin, CA, USA) and visualization with diaminobenzidine (D-5637; Sigma-Aldrich, St. Louis, MO, USA). We included human and nonhuman primate positive and negative control tissues with omitted first-layer antibody. Tissue from all animals showed positive granular cytoplasmic hepatocyte immunolabeling, with varying percentages of involvement and immunolabeling intensity (Technical Appendix Table 2). Immunopositivity was more intense in degenerating and remaining hepatocytes, most often encompassing periportal cord segments and terminal plates (Figure 2, panel D). Necrotic hepatocytes consistently lacked immunoreactivity.

For PCRs, we extracted viral RNA from whole blood (n = 6); serum (n = 1); and liver, kidney, and spleen (n = 4) tissue samples by using a QIAamp RNA Blood Mini Kit (QIAGEN, Valencia, CA, USA), following the manufacturer’s instructions. We amplified virus fragment by using the protocol designed by Drosten et al. (*9*), which targets the 5′ noncoding region (112-bp long) of the genome. All samples were positive; cycle thresholds were 11–26 (cutoff value 38; Technical Appendix Table 2).

Brazil has an established yellow fever national surveillance program (e.g., postmortem NHP studies, vector analyses) focused on early detection of virus circulation, which enables prompt implementation of vaccination and vector control (*8*). Yellow fever diagnosis under this program is achieved by histopathologic, IHC, and PCR results from liver samples of NHPs, performed by reference laboratories (e.g., Adolfo Lutz Institute, São Paulo, Brazil). A multidisciplinary work team and combination of laboratory techniques are essential for a quick, high-quality response that initiates field actions (e.g., vaccination, vector control).

We observed classic histopathologic commonalities with yellow fever–associated disease in humans (midzonal lytic necrosis, apoptotic bodies, steatosis, and scarce paucicellular inflammation) (*10*,*11*). The severity and extent of these lesions probably accounted for severe hepatic failure and death of the animals. The knowledge of naturally occurring yellow fever–associated disease in NHPs gained since the early 1900s is fragmentary (*12*) and mainly limited to *Alouatta* spp. and *Callithrix* spp. NHPs (*6*,*11*). *Alouatta* spp. monkeys are very susceptible to yellow fever; fatal disease has supported a sentinel role for yellow fever virus circulation since the 1930s (*11*). Hepatic lesions of several NHP species, experimentally or naturally infected, are similar to those in humans (*11*). In this study, 1 animal had atypical liver histopathologic features, characterized by massive steatosis with rare midzonal to random single-cell necrosis/apoptosis. Additional liver findings were multifocal microabscesses, probably the result of acute bacterial ascending (intestinal) infection or concomitant septicemia; endothelial necrosis; and attempted hepatic regeneration as suggested by oval cell hyperplasia. The pathologic signature of yellow fever–associated disease in NHPs (≈150 species) is not fully resolved. Ongoing comparisons between yellow fever–associated disease in humans and NHPs may help elucidate convergent and divergent pathogenetic mechanisms and characterize typical and atypical features, thus delineating the pathologic signature in NHPs.

Before the advent of PCR, the method of choice for diagnosing yellow fever was IHC, which remains a highly reliable diagnostic tool (*13*), even in the presence of some autolysis (*14*). In our study and the ongoing outbreak, histopathologic analyses and IHC were vital for successful diagnosis, enabling detection of atypical presentation, paralleling the sensitivity and specificity of PCRs. In a public health context, IHC will greatly aid in yellow fever diagnosis when molecular analysis is not an option, as occurred for 11 of 22 cases in this study. Although RNA extraction from paraffin-embedded tissues is possible, it is not extensively applied and the degradation of RNA may lead to false-negative results.

## Conclusions

During January–July 2017, yellow fever was diagnosed for 150 of 1,000 tested NHPs (15% occurrence) from southern states of Brazil. NHPs were effective yellow fever sentinels and enabled rapid government response (*8*,*15*). Although the Espirito Santo outbreak had damaging socioeconomic and environmental consequences, adequate case conduction and diagnosis may have prevented further human deaths and diminished disease expansion. In contrast, the effects of yellow fever virus spreading among NHPs in the Atlantic forest are expected to be devastating. 

Technical Appendix Histology, immunohistology, and molecular analysis results for 22 nonhuman primates that died of yellow fever, Espirito Santo, Brazil, 2017.
