# Supplementary material for: Outbreak of Yellow Fever among Nonhuman Primates, Espirito Santo, Brazil, 2017
Source: Emerg Infect Dis. 2017 Dec;23(12):2038–41. doi: 10.3201/eid2312.170685 (PMC5708241; doi:10.3201/eid2312.170685)
Supplement: Technical Appendix — Histology, immunohistology, and molecular analysis results for 22 nonhuman primates that died of yellow fever, Espirito Santo, Brazil, 2017. [file 17-0685-Techapp-s1.pdf]

Article DOI: https://doi.org/10.3201/eid2312.170685

Outbreak of Yellow Fever among Nonhuman Primates, Espirito Santo, Brazil, 2017

Technical Appendix

Appendix Table 1. Histologic findings in the livers of 22 nonhuman primates that died of yellow fever, Espirito Santo, Brazil, 2017*

	Necrosis		Councilman bodies		Inflammatory infiltrate			Steatosis				
No.	Intensity	Distribution	Intensity	Distribution	Type	Distribution	Intensity	Type	Distribution	Intensity	Hemorrhage	Other findings
1	+++	Midz/perip	+	Midz/Perip	1,3,4	Sin/Port	+	Ma/Mi	Diff	++	–	–
2	+++	Diff	+	Diff	1,4†	Rand/Sin	++/+	Ma/Mi	Diff	++	–	–
3	+++	Diff	++	Diff	1,3,4†	Sin/Rand	+/+	–	–	–	+ (Rand)	Endothelial necrosis
4	+++	Cent/mid to Diff	++	Diff	1,3,4	Rand/Diff	++	Ma/Mi	Diff	+	++	Endothelial necrosis
5	++	Midz/centr	+++	Midz/Diff	1,3,4†	Sin/Diff	++	–	–	–	+++ (Rand)	Oval cell hyperplasia
6	+++	Diff	++	Diff	1,4	Sin	–/+	Ma	Diff	++	–	Hemosiderosis
7	+++	Diff	++	Diff	1	Sin	+	Ma/Mi	Diff	+	–	–
8	+++	Diff	++	Diff	1,2,3	Diff‡	++	Mi	Diff	+	+ (Rand)	Hemosiderosis
9	+++	Diff	++	Diff	1,3,4	Diff	++	Ma/Mi	Perip	+	–	–
10	+++	Diff	++	Rand/Diff	1,3	Diff	++	–	–	–	++ (Rand)	Endothelial necrosis
11	++	Midz/perip	+	Rand/Diff	1,3,4†	Rand	++	Ma/Mi	Diff	+	–	–
12	+++	Diff	+	Rand	1,3,4	Diff‡	+++	Mi	Diff	+	–	Endothelial necrosis; oval cell hyperplasia
13	++	Cent/midz	+	Midz	1,3	Midz/Port	++	Ma	Perip	++	++ (Midz)	–
14	+++	Diff	+	Rand	1,3,4†	Sin/Rand	+	Mi/Ma	Diff	+	+ (Midz)	Endothelial necrosis
15	++	Midz to Diff	++	Midz/Diff	1,3	Sin/Diff	+	Ma/Mi	Diff	++	+++ (Perip/Diff)	Hemosiderosis; bacterial emboli
16	+++	Diff	+++	Diff	1,3	Sin/Diff	+	Ma	Diff	+	–	–
17	+++	Diff	++	Diff	1,3	Sin/Diff‡	+	Mi/ma	Diff	+	+++ (Cent)	Hemosiderosis
18	+++	Diff	++	Diff	1,3,4	Sin	+	Ma/Mi	Diff	+	+++(Diff)	–

No.	Necrosis		Councilman bodies		Inflammatory infiltrate			Steatosis			Hemorrhage	Other findings
	Intensity	Distribution	Intensity	Distribution	Type	Distribution	Intensity	Type	Distribution	Intensity		
19	+	Rand to diff	++	Diff	1,3,4	Perip	+	Ma	Diff	+	–	Microthrombi
20	+	Midz	+	Midz	1,3,4†	Diff	+	Ma	Diff	+++	+	Hemosiderosis
21	+++	Midz to diff	++	Diff	1,3	Sin/Port	+	Ma/Mi	Diff	+	–	–
22	+++	Diff	++	Diff	1,3	Sin	+	Ma/Mi	Diff	++	–	Hemosiderosis

*Necrosis and steatosis intensity is subjectively measured as follows: + (0%–33% hepatocytes/×200 power field), ++ (34%–66%), and +++ (67%–100%). Councilman (apoptotic) bodies intensity is subjectively measured as follows + (0–5 apoptotic figures/×200 power field), ++ (6%–10%), and +++ (≥11). Inflammatory cell types: lymphocytic (1), plasmacytic (2), histiocytic (3), neutrophilic (4). Cent, centrilobular; diff, diffuse; ma, microvacuolar; mi, microvacuolar; midz, midzonal; perip, periportal; port, portal; rand, random; sin, sinusoidal.
†Rare neutrophilic microabscesses were noted randomly.
‡Rare portal and periportal lymphoid follicles were noted.

Appendix Table 2. Immunohistochemical findings in the liver and results of molecular analysis for 22 nonhuman primates that died of yellow fever, Espirito Santo, Brazil, 2017*

No.	Immunolabeled hepatocytes, %	Immunolabeling intensity	Distribution	PCR cycle threshold value (organ)
1	51–75	++	Diffuse	Not examined
2	76–100	+	Diffuse	Not examined
3	26–50	++	Diffuse	22 (blood)
4	26–50	+++	Diffuse	Not examined
5	26–50	+++	Periportal	23 (blood)
6	76–100	++	Diffuse	19 (liver); 15 (kidney, spleen)
7	51–75	+++	Diffuse	13 (liver); 16 (kidney, spleen)
8	0–25	+	Periportal	13 (liver); 16 (kidney, spleen)
9	26–50	++	Periportal	Not examined
10	26–50	++	Random centrilobular	Not examined
11	76–100	+++	Diffuse	11 (liver); 18 (spleen, kidney)
12	26–50	+	Periportal to diffuse	Not examined
13	51–75	+++	Periportal to centrilobular	12 (blood)
14	0–25	+	Periportal	Not examined
15	26–50	++	Midzonal to periportal	Not examined
16	26–50	+++	Periportal	Not examined
17	0–25	++	Periportal	14 (blood)
18	0–25	++	Periportal	14 (blood)
19	51–75	++	Diffuse	14 (blood)
20	26–50	+++	Periportal to diffuse	26 (serum)
21	51–75	++	Periportal to diffuse	Not examined
22	51–75	+	Periportal	Not examined
